# *Rickettsia* spp. in Seabird Ticks from Western Indian Ocean Islands, 2011–2012

**DOI:** 10.3201/eid2005.131088

**Published:** 2014-05

**Authors:** Muriel Dietrich, Camille Lebarbenchon, Audrey Jaeger, Céline Le Rouzic, Matthieu Bastien, Erwan Lagadec, Karen D. McCoy, Hervé Pascalis, Matthieu Le Corre, Koussay Dellagi, Pablo Tortosa

**Affiliations:** Centre de Recherche et de Veille sur les Maladies Émergentes dans l’Océan Indien, Sainte Clotilde, Réunion Island, France (M. Dietrich, C. Lebarbenchon, C. Le Rouzic, M. Bastien, E. Lagadec, H. Pascalis, K. Dellagi, P. Tortosa);; Université de La Réunion, Saint Denis, Réunion Island, France (M. Dietrich, C. Lebarbenchon, P. Tortosa, A. Jaeger, C. Le Rouzic, M. Bastien, M. Le Corre);; Institut de Recherche pour le Développement, Sainte Clotilde (E. Lagadec, H. Pascalis, K. Dellagi);; Maladies Infectieuses et Vecteurs: Ecologie, Génétique, Evolution et Contrôle, Montpellier, France (K.D. McCoy)

**Keywords:** host specificity, Rickettsia spp., seabird ticks, western Indian Ocean, Rickettsia africae, Amblyomma loculosum ticks, Carios capensis ticks, Rickettsia hoogstraalii, Rickettsia bellii, genetic diversity, geographic range, host, parasites, terrestrial hosts, bacteria

## Abstract

We found a diversity of *Rickettsia* spp. in seabird ticks from 6 tropical islands. The bacteria showed strong host specificity and sequence similarity with strains in other regions. Seabird ticks may be key reservoirs for pathogenic *Rickettsia* spp., and bird hosts may have a role in dispersing ticks and tick-associated infectious agents over large distances.

Rickettsia infections have been explored in many tick species parasitizing a wide variety of animal hosts (*1*). *Rickettsia africae*, the agent of African tick-bite fever, has been detected in the seabird tick *Amblyomma loculosum* (*2*), suggesting a role for seabirds in the maintenance, transmission, and large-scale dispersal of emerging *Rickettsia* spp.

In tropical regions, 2 common and abundant tick species are associated with seabird colonies: the hard tick, *A. loculosum*, and the soft tick, *Carios capensis* (*3*). *Rickettsia* spp. have been documented in these tick species from different areas, although only 1 report is available for tropical areas (*2*). *R. hoogstraalii, R. felis,* and an undescribed species (*Rickettsia* sp. scc49) were detected in *C. capensis* ticks in South Carolina, USA (*4*,*5*). *R. hoogstraalii* was also reported in *C. capensis* ticks from Japan (*6*), raising questions regarding the host and geographic range of *Rickettsia* spp. in seabird ticks.

To better understand the role of seabirds and seabird ticks as reservoirs and dispersers of pathogens, we investigated *Rickettsia* spp. infections in *A. loculosum* and *C. capensis* ticks. These 2 tick species are commonly found on remote tropical islands of the western Indian Ocean. Using molecular detection techniques, we examined variations in detection rates and estimated the genetic diversity of *Rickettsia* spp. between islands and between tick species. 

## The Study

**Figure 1:**
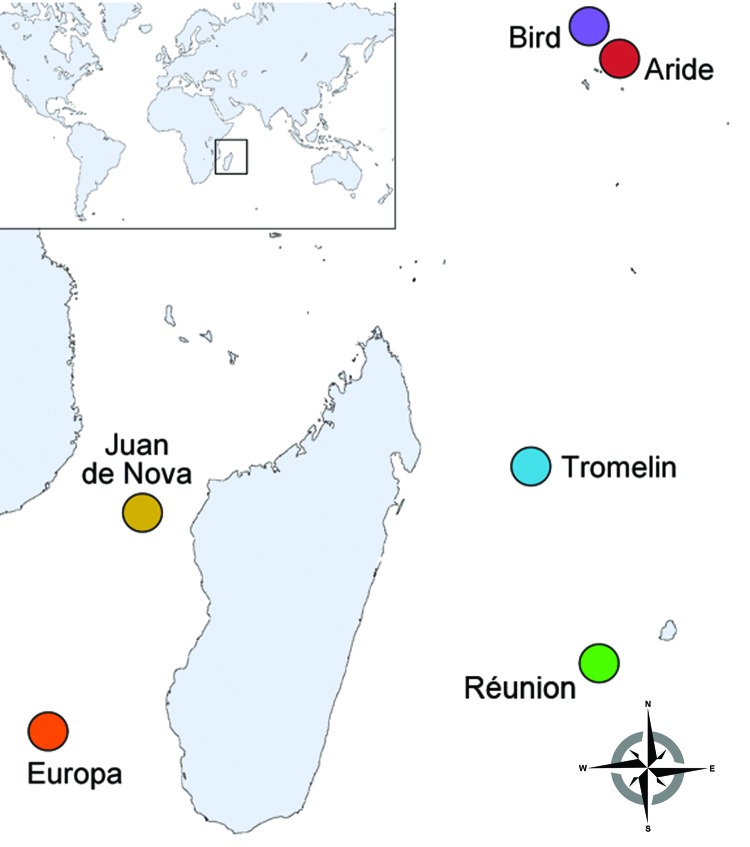
Location of western Indian Ocean islands where tick sampling was conducted among seabird colonies during 2011–2012.

During 2011 and 2012, we collected ticks from seabird colonies on 6 islands of the western Indian Ocean: Aride, Bird, Europa, Juan de Nova, Tromelin, and Réunion Islands (Figure 1; Table). Sample collection on Réunion, Juan de Nova, Tromelin, and Europa Islands was conducted under the approval of the Direction de l'Environnement, de l'Aménagement et du Logement, the Conservatoire du Littoral–Antenne Océan Indien, and the Terres Australes and Antarctiques Françaises. Sample collection on Aride and Bird Islands and their export to Réunion Island were performed with the approval of the Seychelles Bureau of Standards and the Ministry of Environment.

**Table:** Species and numbers of ticks collected during a study of the distribution and host specificity of *Rickettsia* spp. in seabird ticks on 6 remote islands in the western Indian Ocean, 2011–2012

Island, host species	Tick species	No. ticks detected	Detection rate, %, *Rickettsia* spp. (±95% CI)
Aride			
* Puffinus pacificus, Onychoprion fuscatus*	*Amblyomma loculosum*	31	45 (18)
Bird			
* O. fuscatus*	*Carios capensis*	13	8 (15)
	*A. loculosum*	41	37 (1)
Europa			
* O. fuscatus*	*C. capensis*	42	74 (1)
Juan de Nova			
* O. fuscatus*	*C. capensis*	43	16 (1)
Réunion			
* P. pacificus*	*C. capensis*	39	59 (1)
	*A. loculosum*	72	46 (12)
Tromelin			
* Sula sula, S. dactylatra*	*C. capensis*	23	26 (18)
	*A. loculosum*	14	93 (13)
Total		318	45 (5)

**Figure 2:**
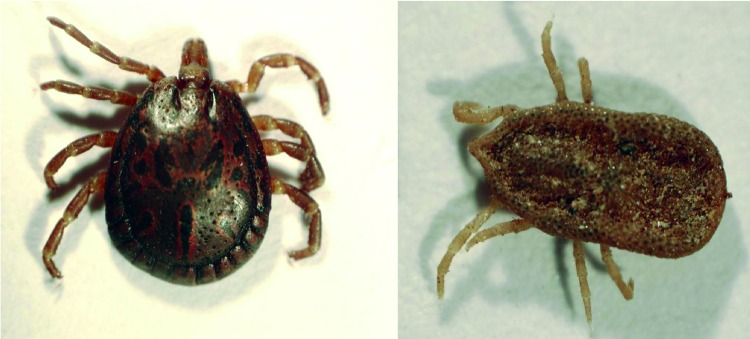
*Amblyomma loculosum* (left) and *Carios capensis* (right) ticks from seabird colonies on western Indian Ocean islands.

All ticks were morphologically identified as *A. loculosum* or *C. capensis* by using standard taxonomic keys (Figure 2). Ticks were individually washed in distilled water and homogenized in Dulbecco modified essential medium or Buffer AVL (QIAGEN, Valencia, CA, USA). Total nucleic acids were extracted by using the EZ1 Viral mini kit v2 and the BioRobot EZ1 System or the QIAmp Viral RNA Mini Kit (all from QIAGEN). We used a commercial cDNA kit (Promega, Madison, WI, USA) to generate cDNAs by reverse transcription. cDNA and PCR, with primers selecting for a 1,064-bp fragment of the citrate synthase–encoding gene (*gltA*), were used to detect *Rickettsia* spp. (*7*). Sequencing of this fragment enabled putative identification of the detected rickettsiae, although complete genotyping is required to fully characterize them (*8*).

Overall, 143 of the 318 ticks tested were positive for *Rickettsia* spp., corresponding to a global detection rate (± 95% CI) of 45% (± 5%) (Table); detection from *A. loculosum* (47% ± 8%) and *C. capensis* (43% ± 8%) ticks was similar (χ^2^ = 0.79, df = 1, p = 0.37). However, detection rates differed significantly among the islands (χ^2^ = 37.96, df = 5, p<0.001). For example, the detection rate was lower in *C. capensis* ticks on Bird (8% ± 15%) and Juan de Nova (16% ± 11%) Islands compared with other islands. The detection rate in *A. loculosum* ticks on Réunion Island was particularly high (93% ± 11%) compared with that on other islands.

**Figure 3:**
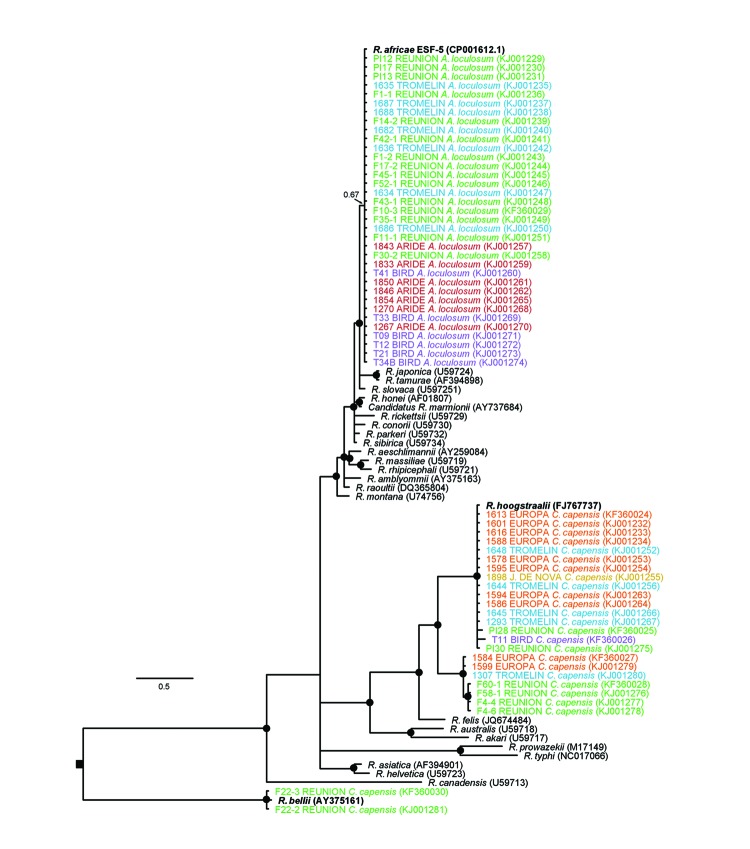
Maximum clade credibility tree for *Rickettsia* spp. detected in seabird ticks (*Amblyomma loculosum* and *Carios capensis*) of the western Indian Ocean as determined on the basis of a 913-bp fragment of the *Rickettsia gltA* gene. The nucleotide substitution model was selected by using the jModelTest 2.1.2 tool (https://code.google.com/p/jmodeltest2/), and Bayesian analyses were performed using MrBayes 3.1.2 (http://mrbayes.sourceforge.net/), with chain lengths of 2 million generations sampled every 1,000 generations. Black dots indicate Bayesian posterior probabilities >0.7. Taxa names are represented by the identification of the sample, the sampling site, and the tick species. GenBank accession numbers are indicated in parentheses. The sequences generated in this study are color-coded according to the geographic origin of samples (see Figure 1) and are accessible in GenBank (accession nos. KF360024–KF360030 and KJ001229–KJ001281). Scale bars indicates nucleotide substitutions per site.

Partial sequencing was performed on the *Rickettsia* spp. *gltA* gene from 60 ticks, including ticks from each island and both species. Phylogenetic analyses revealed 7 haplotypes clustering in 4 well-supported clades; some of the haplotypes corresponded to previously described species (Figure 3). No association was found between *Rickettsia* spp. and collection location (island), but strong host (tick) specificity was observed (Figure 3). All *A. loculosum* ticks were infected with a *Rickettsia* strain showing 100% nt identity with the *R. africae* strain infecting *A. loculosum* ticks in New Caledonia and with the ESF-5 strain isolated from the cattle tick, *A. variegatum*, in Ethiopia (GenBank accession no. CP001612.1). *Rickettsia* spp. in *C. capensis* ticks had 3 well-supported genetic clusters and, thus, were more diverse. The most frequent *Rickettsia* sp. in these ticks (64% of the sequences) was a probable strain of *R. hoogstraalii.* Other haplotypes corresponded to an unknown *Rickettsia sp.* that was closely related to *R. hoogstraalii* and to a probable strain of *R. bellii* (28% and 8% of the sequences, respectively). The haplotype of *R. bellii* was detected only on Réunion Island in 2 *C. capensis* ticks collected within the same nest of the wedge-tailed shearwater (*Puffinus pacificus*).

## Conclusions

We provide evidence of *rickettsia* infection in seabird ticks from 6 islands in the western Indian Ocean. Although the tick infection rate varied among the islands, *Rickettsia* spp. were detected in all studied seabird populations and overall *Rickettsia* infection rates were comparable to those reported for ticks parasitizing terrestrial hosts (*9*).

Phylogenetic analyses revealed a diversity of *Rickettsia* spp. showing strong host specificity. The most frequently detected *Rickettsia* sp. was *R. africae*, the causative agent of African tick-bite fever. *R. africae* was found only in the hard tick, *A. loculosum*. These findings support the hypothesis that *R. africae* is well adapted to this tick host, which may act as a major reservoir for the pathogen (*8*). Moreover, the similarity of *R. africae* sequences from our study with those of strains found in other parts of the world indicates that host shifts may be frequent for this bacterium, facilitating its spread in different tick populations. A better understanding of exchanges between wild and domestic fauna and the possible emergence of *R. africae* at regional levels will require further investigations of the link between *R. africae* in seabird ticks and in cattle ticks (*10*).

We detected 2, possibly 3, *Rickettsia* spp. in *C. capensis* ticks; this genetic diversity was higher than that detected in *A. loculosum* ticks. In accordance with previous descriptions (*4*,*6*), *R. hoogstraalii* was the most common *Rickettsia* species infecting *C. capensis* ticks, confirming that this tick species likely represents a major host reservoir for this bacterium. An unclassified rickettsial lineage, genetically related to *R. hoogstraalii*, was also identified in *C. capensis* from Réunion, Europa, and Tromelin Islands, showing that 2 distinct *Rickettsia* spp. cocirculate in these tick populations. Additional sampling and further genetic characterization (*8*) are required to better describe the distribution of this lineage and its relationships to *R. hoogstraalii*.

*R. bellii* has mainly been documented in the Americas, but recent studies have detected the presence of closely related *R. bellii* strains in Australia (*11*) and Europe (*12*). Although we detected *R. bellii* in only 2 ticks on 1 island, our study findings show the potential for broad distribution of this bacterium because of its large arthropod host range (*13*). The *R. bellii* genome has been completely sequenced (*13*), but current knowledge on its epidemiology and pathogenicity in humans remains to be evaluated. In addition, our study was restricted to analysis of the *gltA* gene, so additional genotyping studies are needed to fully characterize the *Rickettsia* spp. that we identified (*8*).

The presence of *Rickettsia* spp. on remote islands indicates a possible role of bird hosts in the dispersal of ticks and their associated infectious agents over large distances (*14*,*15*). An understanding of the historic colonization and current population structure of *A. loculosum* and *C. capensis* ticks, together with knowledge of bird migratory patterns, is required to properly assess the risk for emergence of *Rickettsia* spp. on these islands.
